# Flow analysis-solid phase extraction system and UHPLC-MS/MS analytical methodology for the determination of antiviral drugs in surface water

**DOI:** 10.1007/s11356-024-34466-5

**Published:** 2024-07-30

**Authors:** Karolina Mermer, Emilia Jas, Justyna Paluch, Aneta Woźniakiewicz, Michał Woźniakiewicz, Paweł Miśkowiec, Petr Chocholouš, Hana Sklenářová, Joanna Kozak

**Affiliations:** 1 Doctoral School of Exact and Natural Sciences, Jagiellonian University, Łojasiewicza 11, 30-348 Kraków, Poland; 2 Department of Analytical Chemistry, Faculty of Chemistry, Jagiellonian University, Gronostajowa 2, 30-387 Kraków, Poland; 3 Department of Environmental Chemistry, Faculty of Chemistry, Jagiellonian University, Gronostajowa 2, 30-387 Kraków, Poland; 4 Department of Analytical Chemistry, Faculty of Pharmacy in Hradec Králové, Charles University, Heyrovského 1203/8, 500 03 Hradec Králové, Czech Republic

**Keywords:** Antiviral drugs, Solid phase extraction, Flow analysis, Water analysis, UHPLC-MS/MS, Emerging contaminants

## Abstract

An automated flow analysis-solid phase extraction (FA-SPE) system and methodology of ultra-high-performance liquid chromatography with tandem mass spectrometry (UHPLC-MS/MS) analysis were developed for the determination of selected antiviral drugs (acyclovir, amantadine, rimantadine, and oseltamivir) in water samples. The proposed FA-SPE approach enables the integration of various extraction stages and elimination of the sample evaporation step and offers individual customisation of SPE parameters, inter alia sample, and eluate flow rate and volume. Using the developed FA-SPE procedure, e.g. a 100-fold preconcentration of the target analytes in 1 h was achieved. A method for chromatographic analysis was also developed to determine the selected antiviral drugs in combination with the use of the FA-SPE system. The developed FA-SPE UHPLC-MS/MS method was validated including the determination of linearity of analytical graphs, limits of detection (5.5–99.9 pg mL^−1^) and quantification (18.3–329.8 pg mL^−1^), intra-day (1.8–8.3%) and inter-day (3.0–9.2%) precision, recovery (95.6–105.3%), and matrix effects (− 12.9 to 13.2%). The proposed method was successfully applied to analyse tap, drinking, and river water samples, revealing the presence of amantadine at a concentration of 40.1 pg mL^−1^ in one sample. The environmental impact of the developed FA-SPE sample preparation procedure was also assessed using the AGREEprep metric tool and compared with five other literature methods, achieving the most sustainable outcome.

**Graphical abstract:**

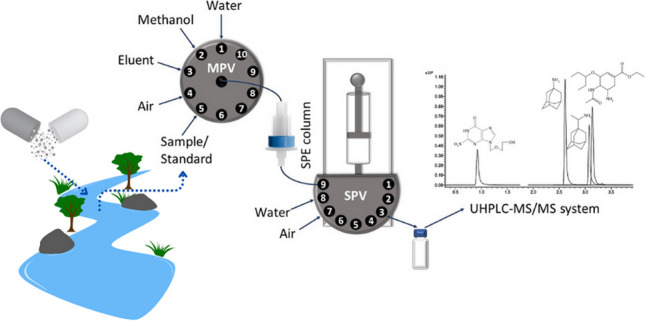

**Supplementary Information:**

The online version contains supplementary material available at 10.1007/s11356-024-34466-5.

## Introduction

In the twenty-first century, antiviral drugs have become essential tools in the fight against the myriad viral infections afflicting human populations worldwide. These pharmaceuticals have transformed previously lethal diseases into treatable conditions, saving countless lives. Nevertheless, their widespread usage is not without consequences (Nannou et al. 2020; Eryildiz et al. 2022). As antiviral drugs are increasingly prescribed and consumed, the hazards associated with their abuse are growing, causing serious environmental concerns, particularly for water reservoirs. The release of these drugs into surface water systems through wastewater discharges and inappropriate disposal poses challenges. The aquatic environment becomes a habitat where antiviral drugs can accumulate and persist, potentially disrupting fragile ecosystems and encouraging the development of drug-resistant viruses among aquatic organisms (Couto et al. 2019; Krasucka et al. 2022).

Nowadays, there are 13 groups of 90 approved antiviral drugs categorised according to their mechanism of action (Nannou et al. 2020). These antiviral drugs are used to treat several hundred known human viral infectious diseases, such as among others influenza and HIV. The most widely administered antiviral drugs, which were analysed in the study, are acyclovir and oseltamivir (Vardanyan and Hruby 2016; Nannou et al. 2019; Kausar et al. 2021). Their widespread use in clinical practice, available in a variety of formulations, including oral tablets, topical creams, and intravenous preparations, has significantly improved the quality of life for those suffering from these viral diseases. Therefore, they are one of the antiviral drugs with the highest number of analytical assay methods available in the literature (Eryildiz et al. 2022). The other examples of selected antiviral drugs that are equally important, but there are only a few assay methods for them, are amantadine and rimantadine (Vardanyan and Hruby 2016; Kausar et al. 2021).

Identifying the presence of antiviral drugs in surface waters such as rivers and lakes commonly requires a combination of analytical techniques, as their concentrations are usually low, ranging from a few pg mL^−1^ to a few ng mL^−1^ (Jain et al. 2013; Nannou et al. 2019). Firstly, in most cases, extraction is necessary to preconcentrate and isolate target compounds from a large volume of water samples. The most commonly used extraction method for this purpose is solid phase extraction (SPE), performed both on-line (Marasco Júnior et al. 2021) and off-line (Azuma et al. 2019; He et al. 2022; K'oreje et al. 2016). Next, high-performance liquid chromatography coupled with mass spectrometry (HPLC-MS) (Takanami et al. 2012; Funke et al. 2016) or tandem mass spectrometry (HPLC-MS/MS) (Azuma et al. 2017; K'oreje et al. 2016; Söderström et al. 2009) is commonly used for quantitative analysis of antiviral drugs due to their high sensitivity and specificity. Integrating these methods enables comprehensive monitoring of antiviral drug residues in surface waters, which is essential for assessing potential environmental impacts and ensuring water quality. It should be pointed out that in the literature, there are still very few analytical methods for the determination of antiviral drugs in surface water/wastewater, and therefore, it is necessary to develop novel approaches (Eryildiz et al. 2022).

The use of flow analysis techniques in the analytical procedure greatly increases automation and improves analysis performance. Through appropriate modifications of used systems, it is possible to perform chemical reactions with sample components, separate sample components, enrich analytes using lower consumption, smaller amounts of solvents, and reduce analysis time (Melchert et al. 2012). This meets the principles of Green Analytical Chemistry (Tobiszewski et al. 2010) in terms of conducting not only the analysis but also sample preparation. The use of flow analysis can also improve the validation parameters of the method, especially precision (Melchert et al. 2012). Employing a flow system to conduct SPE enables the entire process to be automated, thereby reducing the influence of the human factor on the analysis and, at the same time, reducing the risk of operator exposure to hazardous chemicals. Using such a solution also allows adaptation of the conditions of the system to the requirements of the analyst. In addition, the system can become an on-line system once instrumental conditions enable it.

Numerous methods for assessing the environmental friendliness of analytical protocols are available in the literature. These methods can be categorised, for example, according to whether they evaluate the entire analytical method (Gałuszka et al. 2012; Nowak and Kościelniak 2019; Pena-Pereira et al. 2020; Płotka-Wasylka and Wojnowski 2021) or only the sample preparation step (Wojnowski et al. 2022). In this study, the sample preparation methods’ greenness was evaluated using the Analytical Greenness Metric for Sample Preparation (AGREEprep) (Wojnowski et al. 2022). AGREEprep is a free application that enables the assessment of the greenness of a sample preparation method based on ten categories. It is possible to differentiate the importance of the categories by assigning them weights (from 1 to 5). The higher the final evaluation score (closer to 1.0), the greener the sample preparation method.

The aim of the study was to develop a procedure for the determination of antiviral drugs, selected from different groups (acyclovir, amantadine, rimantadine, and oseltamivir), in surface water samples using an originally designed, automated flow analysis-solid phase extraction (FA-SPE) system and ultra-high-performance liquid chromatography with electrospray ionisation and tandem mass spectrometry (UHPLC-MS/MS). During the literature review, no publication was found in which the selected compounds were determined simultaneously in surface water. Therefore, beyond the use of the proposed FA-SPE system for sample preparation, a chromatographic method was also developed purposely for the determination of these analytes. Both the FA-SPE system performance and the chromatographic method were optimised. The developed FA-SPE UHPLC-MS/MS method was validated and subsequently employed for the determination of antiviral drugs in surface (river) water samples. The developed original approach to sample preparation was also evaluated in terms of environmental impact.

## Materials and methods

### Chemical standards and reagents

The analytical standards of acyclovir (ACV; ≥ 99%), amantadine hydrochloride (AMA; > 99%), rimantadine hydrochloride (RIM; > 99%), and oseltamivir phosphate (OS; > 99%) were purchased from Sigma-Aldrich Ltd. (St. Louis, MO, USA). Acetonitrile and methanol, both hypergrade for LC-MS LiChrosolv®, were obtained from Sigma-Aldrich Ltd. (Supelco®; St. Louis, MO, USA). Mobile phase additive formic acid (≤ 98%, LC-MS grade) was obtained from Merck (Darmstadt, Germany). Deionised water (18.2 MΩ cm^−1^, TOC < 5 ppb) was produced using the Milli-Q Plus system from Millipore (Burlington, MA, USA). Once diluted, sodium hydroxide (an ampoule of Titrisol® 1 mol L^−1^; Merck, Darmstadt, Germany) and hydrochloric acid (fuming 37%; Merck, Darmstadt, Germany) were used to adjust the pH. Matrix reference material (MRefM) of drinking water EnviroMAT EP-H Drinking Water was purchased from SCP SCIENCE (Montreal, Canada).

The stock solutions of the AMA, RIM, and OS at 10 mg mL^−1^ were prepared by dissolving, respectively, 12.41 mg of amantadine hydrochloride, 12.03 mg of rimantadine hydrochloride, and 13.14 mg of oseltamivir phosphate (V), each in 1 mL of methanol. Considering the highly poor solubility of ACV in methanol, a 1 mg mL^−1^ stock solution of acyclovir was prepared by weighing and dissolving 1.00 mg of the standard in 1 mL of water. Further dilutions of all standard solutions were prepared in methanol. A mixed standard solution of the analytes prepared in methanol at 10 µg mL^−1^ was used for preparing working solutions and spiked samples. A mixed standard solution of the analytes was prepared in methanol at 1 µg mL^−1^ for optimising mass detector parameters.

### Instrumentation

The studies were conducted using an original, developed off-line flow analysis-solid phase extraction system (FA-SPE) and an ultra-high-performance liquid chromatography-electrospray ionisation tandem mass spectrometry (UHPLC-MS/MS) as a chromatographic separation and detection system, respectively.

### Flow analysis-solid phase extraction system

The extraction of the analytes was performed using an originally designed flow analysis system consisting of a syringe pump integrated with a nine-position selection valve (SPV) equipped with a 5 mL syringe (Seattle, WA, FIAlab, USA), ten-position selection valve (MPV; VICI Valco Instruments, Houston, TX, USA), PTFE tubing (before SPE column: 100 mm, 0.8 i.d.; after SPE column: 40 mm, 0.8 mm i.d.), and a small extraction column (Oasis HLB Plus Light Cartridge 30 mg, 30 µm, cat. number: 186005125, Waters, Milford, MA, USA) adapted to the use in a flow analysis system. The operation of the pumps was controlled by FIAlab for Windows 5.11.19. Each extraction procedure, besides testing the performance of the employed column, was conducted using a fresh column.

### UHPLC-MS/MS instrument

The separation and quantification of analytes were performed using an UHPLC-MS/MS system − model LCMS-8045 (Shimadzu, Kyoto, Japan) equipped with an electrospray ionisation source (ESI). The LCMS-8045 comprises a Nexera XP UHPLC unit and a triple quadrupole mass spectrometer. The Nexera XP UHPLC system includes a high-pressure binary pump, on-line degasser, UV/VIS detector, autosampler, and column oven. The UPHLC system and a mass spectrometer were connected by a dual-position valve, enabling the direction of effluent to MS or waste to avoid detector contamination. The UPHLC-MS/MS data were collected by LabSolutions 5.118 software and processed by LabSolutions Insight 4.0 software (Shimadzu, Kyoto, Japan).

### Sample collection

The surface river water was collected from rivers in southern Poland − river water samples 1 and 2 were collected from urban areas, and river water samples 3–5 were collected from the countryside. Samples were collected into 1 L amber glass bottles and stored in a refrigerator at + 4 °C. Water samples were not kept longer than 4 days. Directly before analysis, the water was filtered using syringe filters with a pore size of 0.45 µm. Tap water was collected in the laboratory into a 1 L amber glass bottle and then analysed the same day. Matrix reference material (MRefM) of drinking water was prepared according to the manufacturer’s recommendations, diluting 10 mL of MRefM with ultrapure water to obtain a 1 L of sample. Such a sample was stored in a 1 L amber glass bottle to be analysed on the same day it was prepared.

## Results and discussion

The preliminary studies included the selection of both UHPLC and MS/MS operating conditions and the selection of sample preparation FA-SPE system conditions for analysis, including the selection of the SPE column and eluent, the development of operating conditions for the FA-SPE system, the selection of pH of the sample, the volume of the extracted sample, and verification of the possibility of multiple use of the extraction column.

### UHPLC-MS/MS analysis

To perform the chromatographic separation step, an Ascentis Express C18 chromatography column (100 mm × 2.1 mm; 2.7 µm superficial porous particles; Sigma-Aldrich, St. Louis, MO, Germany) was employed based on literature data (Azuma et al. 2017, 2019). The column was thermostated at + 40 °C. The mobile phase was prepared by mixing of 0.1% aqueous formic acid solution (A) and acetonitrile (Azuma et al. 2017, 2019) (B) in gradient mode (component B: 3% from 0 min and increasing to 95% by 7 min, maintaining these conditions for 1 min, returning to 3% in 0.1 min, and continuing these conditions until 11 min). These gradient elution conditions were used to eluate all analytes and possible matrix residues. The mobile phase flow rate was 0.4 mL min^−1^, and the sample injection volume was 0.1 µL. The temperature of the autosampler was set at + 4 °C. Chromatograms of the tested compounds were registered in appropriately selected time windows (compound retention time ± 0.75 min). The selected conditions allowed complete separation of all analytes to be achieved in a short time of 3.5 min. The example chromatogram obtained during the analysis is shown in Fig. S1 (Supplementary materials).

The mass spectrometer was operated in MRM (multiple reaction monitoring) mode during the analysis. The MRM transitions were optimised for the detection and quantification of the analytes. The monitored ions for each target compound were selected by injection of standard solutions of each compound (1 µg mL^−1^) into the MS operating in full scan mode (from 150 to 1000 m*/z*). According to the results of the performed injections, it was decided that subsequent measurements would be conducted in positive ionisation mode. The assay of investigated compounds was performed to define three (for ACV) or four (for AMA, RIM, and OS) MRM transitions per compound. For each compound, the most intense transition (from precursor ion to product ion) was selected for quantitative signal, and the others were used for confirmation. During the optimisation of the MRM transitions, the optimal collision energy (CE), Q1 Pre Bias, and Q3 Pre Bias were selected to maximise the signal response for each ion. The interface conditions for mass spectrometer measurements are described below: the ESI interface temperature at 300 °C, desolvation line (DL) temperature at 250 °C, nebulising gas (N_2_) flow at 3 L min^−1^, drying gas (N_2_) flow at 10 L min^−1^, and heating gas (zero air) flow at 10 L min^−1^. The MRM transitions and optimisation results of target compounds are summarised in Table 1.

**Table 1:** Physicochemical parameters and mass spectrometry parameters for investigated antiviral drugs

Analyte	Abbreviation	Physicochemical properties				Retention time, min	Q1^b^: Precursor *m/z*	Q3^b^: Fragment *m/z*	CE, eV	Q1 Pre Bias^c^, V	Q3 Pre Bias^d^, V
		Molecular weight, g mol^−1^	pK_a_ (acidic)^a^	pK_a_ (basic)^a^	logP^a^						
Acyclovir	ACV	225.20	2.27	9.25	− 1.9	0.933	226.20	152.05	− 13.0	− 17.0	− 29.0
							226.20	135.00	− 29.0	− 17.0	− 26.0
							226.20	110.00	− 31.0	− 18.0	− 18.0
Amantadine	AMA	151.25	-	10.58	2.4	2.626	152.25	135.10	− 18.0	− 11.0	− 24.0
							152.25	107.10	− 27.0	− 11.0	− 21.0
							152.25	93.10	− 30.0	− 11.0	− 17.0
							152.25	79.00	− 32.0	− 11.0	− 14.0
Rimantadine	RIM	179.30	-	10.43	2.6	3.087	180.30	163.20	− 14.0	− 10.0	− 11.0
							180.30	80.90	− 25.0	− 14.0	− 30.0
							180.30	107.00	− 28.0	− 11.0	− 21.0
							180.30	120.90	− 25.0	− 19.0	− 11.0
Oseltamivir	OS	312.40	-	7.70	1.1	3.149	313.40	166.10	− 16.0	− 12.0	− 11.0
							313.40	225.15	− 10.0	− 12.0	− 15.0
							313.40	208.10	− 13.0	− 23.0	− 22.0
							313.40	120.05	− 28.0	− 23.0	− 21.0

*pK*_*a*_ negative base-10 logarithm of the acid dissociation constant, *logP* partition coefficient, *CE* collision energy

^a^Values were obtained from PubChem database (https://pubchem.ncbi.nlm.nih.gov/)

^b^The pairs of MRM transitions—first MRM was used in quantitative aim, the others were used as qualifiers

^c^Voltage promotes the ionisation of the precursor ion

^d^Voltage promotes the ionisation of the product ion

### FA-SPE system and sample preparation procedure

**Fig. 1:**
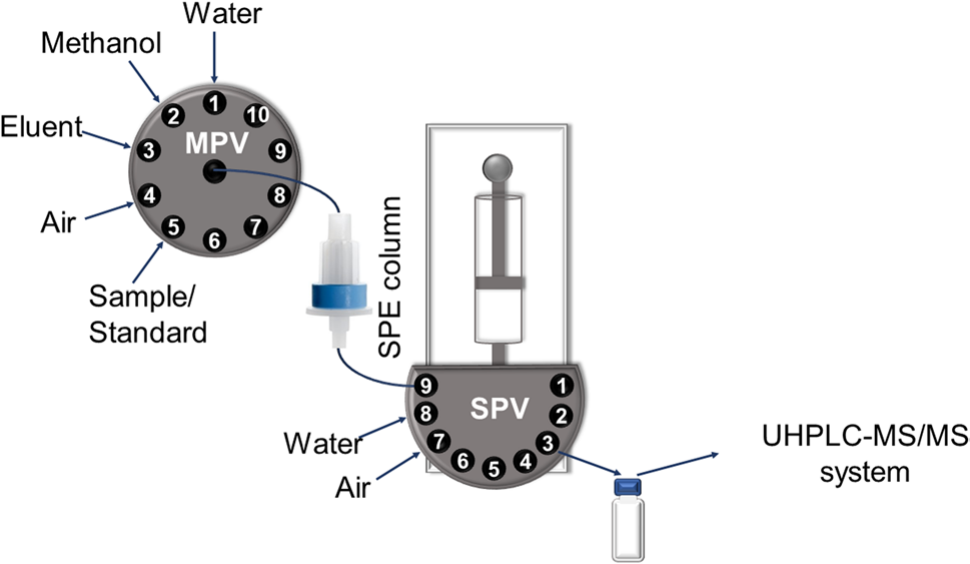
Scheme of the developed FA-SPE system (MPV, ten-position selection valve; SPV, nine-position selection valve)

Solid phase extraction was used for analyte enrichment and sample purification. The flow analysis system developed to perform the SPE procedure is presented in Fig. 1. It is equipped with a SPE column, a ten-position selection valve allowing the introduction of appropriate solutions or air into the extraction column, and a syringe pump with a nine-position selection valve determining the direction of discharge of the syringe content.

Based on the literature review, a SPE column with Oasis HLB sorbent was selected for the study (Azuma et al. 2017; Ghosh et al. 2010; K'oreje et al. 2016). To facilitate the integration of the column directly in a flow system, a column in cartridge format containing 30 mg of sorbent was selected. Also, this sorbent mass and design of the column were chosen to minimise backpressure and enable efficient elution with a small eluent volume in the flow system. The selected Oasis HLB is water-wettable sorbent designed for the extraction of a wide range of acidic, basic, and neutral compounds from a wide variety of matrices and allows operation over a broad pH range from 0 to 14. Each extraction procedure, besides testing the performance of the employed column, was performed using a fresh column. The process of replacing the SPE column is convenient, easy, and quick. The design of the column itself enables its connection to the system through the use of tubing ended with ferrules without rifling secured with PTFE tape. This solution is simple and yet sufficient for performing efficient extraction.

Methanol and acetonitrile were tested as eluents. These solvents were used in pure form and a 1:1 mixture (methanol:acetonitrile) during testing. The highest extraction efficiency (85–104%) was achieved using pure methanol. The extraction efficiency, expressed as a percentage, was defined as the ratio of the determined analyte concentration to the expected analyte concentration in the sample. The application of this solvent as an eluent is also recommended by the manufacturer of the Oasis HLB sorbent used during the study. Although the use of methanol as an eluent involves some drawbacks, e.g. toxicity, negative environmental impact, and equipment hazards, it is widely used in solid phase extraction (Azuma et al. 2017; Azuma et al. 2019; Ghosh et al. 2010; He et al. 2022; K'oreje et al. 2016; Vergeynst et al. 2015). This also prejudged its use in this study.

Based on the recommendations of the manufacturer, literature data (Azuma et al. 2017, 2019), and the authors’ experience concerning flow-based systems, the sample and eluent flow rates through the column of 1 mL min^−1^ were selected. This is the optimal solvent/sample/eluent flow rate for the loading and elution steps considering also the time of analysis.

A further step in the study was to select the appropriate volume of the analysed water sample and the eluted sample volume. For this aim, 50 mL tap water samples containing 500 pg mL^−1^ of analysed drugs were prepared. Measurements were performed for four series, with three samples in each one. In each series, the defined volume of a sample (${V}_{S}$) was subjected to the FA-SPE procedure and eluted with a defined volume of methanol (${V}_{E}$). The enrichment factor was calculated as $EF=\frac{{V}_{S}}{{V}_{E}}$. The extractions with 50-fold (50/1), 75-fold (75/1), 100-fold (50/0.5), and 150-fold (75/0.5) enrichment factors were tested. The study revealed satisfactory extraction efficiencies for analytes ranging from 90 to 110% for all these tested options. Considering the performance of the syringe pump used in the developed system and to obtain the highest possible preconcentration, because it was decided to eliminate the sample evaporation step of standard SPE procedure, an eluate volume of 0.5 mL was selected. This was the minimal volume that ensured reproducible recovery results with the use of the applied syringe pump. With regard to the analysis time and the possible concentration of analytes in surface water samples, it was decided to conduct subsequent stages of the study for 100-fold preconcentration of analytes. Respecting that the sample flows through the column at a rate of 1 mL min^−1^, it can be calculated that the extraction of 50 mL of a sample requires approximately 1 h, and these conditions were selected for publication purposes. Larger sample volumes such as 75 mL or 100 mL required a longer time.

The steps of the sample preparation procedure in the FA-SPE system were based on the procedure performed in conventional SPE systems. To improve and simplify the procedure, the step of air drying of the sorbent was reduced to a minimum and performed reproducibly under the same conditions, directly in the system, while the solvent evaporation and reconstitution steps were eliminated. All the extraction steps were executed in the developed system in an automated manner.

The proposed FA-SPE procedure consisted of the following steps: (1) conditioning the column with methanol and water, (2) introducing the sample, (3) flushing the column with water, (4) drying the column with air, (5) flushing the syringe twice with water and individually with air, and (6) eluting the analyte with methanol. These steps of the proposed procedure are summarised in Table 2.

**Table 2:** The final procedure for analytes extraction using a developed FA-SPE system

Step	MPV position	SPV position	SP flow rate, mL min^−1^	Volume, mL	Solution	Action
(1) Conditioning of the column						
1	2	9	1.0	1.5	Methanol	Aspirating
2	-	1	12.0	1.5	Methanol	Emptying
3	1	9	1.0	1.5	Water	Aspirating
4	-	1	12.0	1.5	Water	Emptying
(2) Introducing the sample^a^						
5	5	9	1.0	5.0	Sample	Aspirating
6	-	1	12.0	5.0	Sample	Emptying
(3) Flushing the column						
7	1	9	1.0	1.0	Water	Aspirating
8	-	1	12.0	1.0	Water	Emptying
(4) Drying the column						
9	4	9	1.0	1.0	Air	Aspirating
10	-	1	12.0	1.0	Air	Emptying
(5) Flushing the syringe						
11	-	8	12.0	2.0	Water	Aspirating
12	-	1	12.0	2.0	Water	Emptying
13	-	8	12.0	2.0	Water	Aspirating
14	-	1	12.0	2.0	Water	Emptying
15	-	7	12.0	2.0	Air	Aspirating
16	-	1	12.0	2.0	Air	Emptying
(6) Elution of the analytes						
17	3	9	1.0	0.5	Methanol	Aspirating
18	4	9	1.0	0.5	Air	Aspirating
19	-	3	1.0	0.5	Eluate	Emptying
20	-	1	12.0	0.5	Air	Emptying

*MPV* multi-position selection valve, *SPV* syringe pump selection valve, *SP* syringe pump

^a^These two steps were repeated ten times (total 50 mL of the sample)

In the developed protocol, to prepare the SPE column, 1.5 mL of methanol followed by 1.5 mL of ultrapure water was passed through it. Then, 50 mL of the water sample was introduced onto the column (in ten repetitions, as the syringe capacity was 5 mL), and the column was flushed with 1 mL of ultrapure water. After that, the column and tubes were dried using 1.0 mL of air to remove any excess water. The pump syringe was flushed and dried to remove residues of the extracted sample. Afterwards, the analytes were eluted using 0.5 mL of methanol. The flow rate was maintained at 1 mL min^−1^ during each extraction step. The developed procedure lasted about 1 h; it allows 50 mL of sample to be preconcentrated to 0.5 mL (100 times) at an eluent and sample’s flow rates of 1 mL min^−1^. Finally, the obtained eluates were analysed using a UHPLC-MS/MS system.

The developed easily adaptable FA-SPE system allows individual customisation of many parameters, including the volume of the analysed sample and the volume of the eluate, as well as the flow rate of the sample and other fluids through the column. Additionally, it should be also noted, that ten-position and nine-position selection valves, applied in the FA system, can also be employed as an autosampler and fraction collector, respectively, to enable setting up several samples and performing their extraction consecutively on the same extraction column, in an automated, operator-independent manner. The vacant positions on the ten-position valve could be used as positions for aspirating successive samples − an autosampler. Meanwhile, the nine-position valve at the syringe pump could be employed as a fraction collector − the spare positions at the valve can be supplied with tubes that will appropriately discharge the obtained eluates, after extracting successive samples, from the syringe into properly capped vials.

### Selection of the pH of analysed samples

**Fig. 2:**
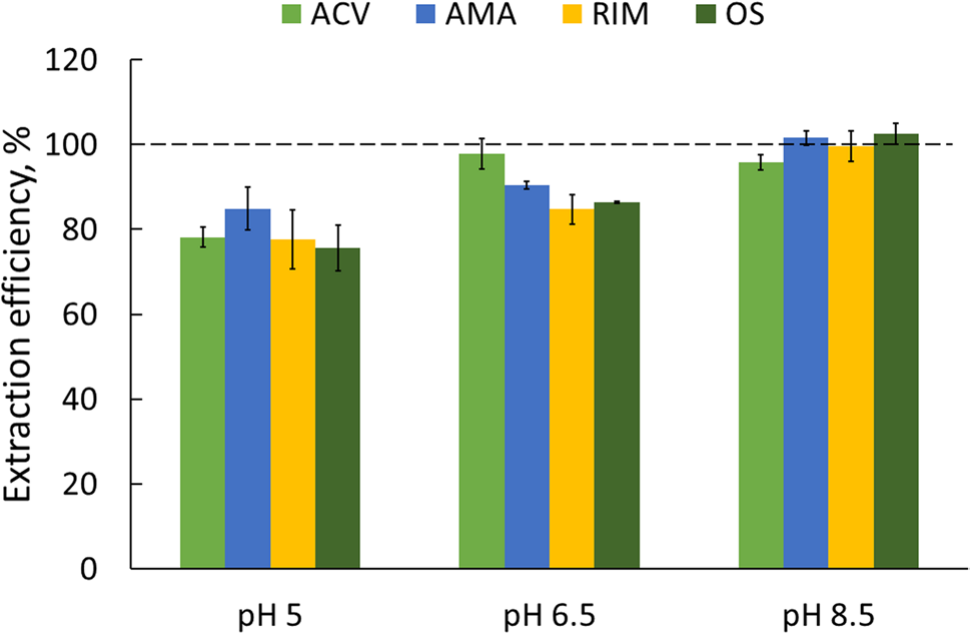
Effect of the sample pH on extraction efficiency (bars show the standard deviation; *n* = 9, three samples, three injections)

A study of the effect of the pH of the analysed sample on the extraction efficiency was performed, and the obtained results are shown in Fig. 2. For the study, three pH values − 5, 6.5, and 8.5 were selected, as drinking or surface water samples commonly present a pH in this range. Tap water samples of 50 mL and a concentration of 500 pg mL^−1^ tested drugs were analysed. The volume of the eluate was 0.5 mL.

The best results were found for a sample pH of 8.5, as the extraction efficiency of all the analytes was almost 100% at this pH. The literature also provides information on conducting solid phase extraction of antiviral drugs from water samples with a pH value comparable to the pH used in this work (Prasse et al. 2010).

### Testing of the SPE column performance

**Fig. 3:**
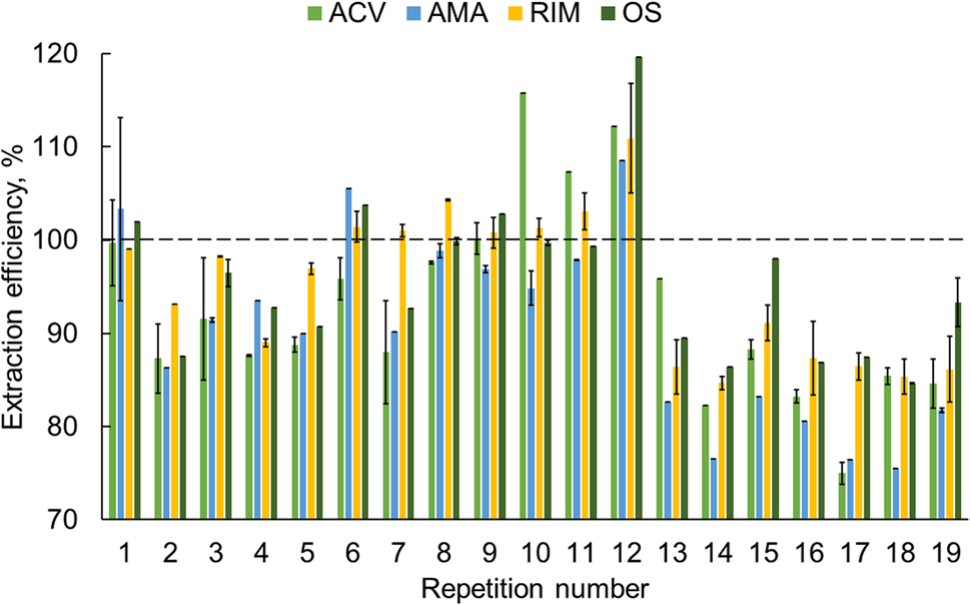
Performance study of an extraction column (bars show the standard deviation; *n* = 3, one sample, three injections)

During this research, it was decided to test the multiple extraction performance of the used columns. Tap water samples of 50 mL and a concentration of 500 pg mL^−1^ tested drugs were extracted. The volume of the eluate was 0.5 mL. For this study, a single SPE column was used to perform 19 consecutive extractions. The results of the study (with error bars showing the appropriate standard deviation) are shown in Fig. 3.

As shown in Fig. 3, the extraction efficiency for all analytes ranges from approximately 90 to 120% until the eleventh repetition, then increases before decreasing significantly in subsequent repetitions. The obtained results indicate that the SPE column can be used multiple times (up to 11) for extracting analytes from water samples, nevertheless, to avoid any false-positive results during the method development, a fresh column was used for the extraction of each sample in this study. The column extraction efficiency study conducted reveals the prospect of increasing the greenness by multiple uses of the extraction column. Furthermore, it is important to highlight that the multiple use of the extraction column implies the development of a sufficient procedure for flushing the sorbent between consecutive sample extractions to avoid carryover effects. In this study, washing with 1.5 mL of methanol followed by 1.5 mL of water proved to be effective.

### Method validation

Validation of the developed method was performed (ICH Harmonised Guideline, 2022) for the determination of the following parameters: linearity, limit of detection (LOD), limit of quantification (LOQ), repeatability and reproducibility expressed as inter-day and intra-day precision, recovery, and matrix effect. The analysis was conducted using ultrapure water samples (50 mL) spiked with the investigated analytes at the following concentrations: 5.0, 10.0, 50.0, 500.0, 1000.0, 3500.0, 7500.0 pg mL^−1^. During the study, the external standard calibration method was used. The calibration curves were created by fitting an unweighted linear regression model to the received data series. In this research, the analytical signal was the area under the peaks obtained for the analytes.

The limit of detection and quantification were determined from the signal-to-noise ratio obtained for the standard sample at the lowest drug concentration (5.0 pg mL^−1^).

Another investigated validation parameter of the method was precision. This parameter was calculated for each of the tested compounds at three concentration levels: low (50.0 pg mL^−1^), medium (750.0 pg mL^−1^), and high (2500.0 pg mL^−1^). The precision was established for the resulting concentrations of the analytes tested. The intra-day precision of the method for each concentration was calculated from triplicate measurements for three samples prepared separately on the same day (*n* = 3). Inter-day precision was determined from measurements performed over 3 days (*n* = 9). In both cases, precision was expressed as a coefficient of variation (CV, %).

The recovery of the method (R, %) was determined for each analyte based on triplicate measurements for three independent samples at three concentration levels (50.0, 750.0, 2500.0 pg mL^−1^). This parameter was calculated using the following formula: $R=\frac{{c}_{i}-{c}_{x}}{{c}_{0}}\cdot 100\%$, where $R$ is the recovery, ${c}_{i}$ is the analyte concentration determined in the sample after spiking, ${c}_{x}$ is the analyte concentration determined in the sample, and ${c}_{0}$ is the concentration of analyte (added) in the spiked sample.

The matrix effect (ME) was determined for all of testes water samples. The matrix effect was calculated based on Matuszewski’s work (Matuszewski et al. 2003) with a slight modification, where a value of matrix effect > 0% indicates ion suppression and a value < 0% indicates ion enhancement. The matrix effect was investigated for each of the listed matrices at three concentration levels: 5.0, 50.0, and 350.0 ng mL^−1^, and was calculated using the below equation: $ME=\frac{{{c}_{z}-c}_{y}}{{c}_{z}}\cdot 100\%$, where ${c}_{y}$ is the analyte concentration determined in the water extracts spiked with standards after extraction and ${c}_{z}$ is the analyte concentration determined in standard solutions (in pure methanol, without extraction). The results of method validation are summarised in Table 3.

**Table 3:** Results of method validation for the developed method; details in the text

Analytical parameter		Analyte			
		ACV	AMA	RIM	OS
Linear range, pg mL^−^^1^		329.8 to 7500.0	27.3 to 7500.0	37.4 to 7500.0	18.3 to 7500.0
Coefficient of determination, *r*		0.997	0.999	0.999	0.998
LOD, pg mL^−^^1^		99.9	8.3	11.3	5.5
LOQ, pg mL^−^^1^		329.8	27.3	37.4	18.3
CV (intra-day, *n* = 3), %	50 pg mL^−1^	-	5.2	7.1	8.3
	750 pg mL^−1^	4.4	3.1	2.7	3.2
	2500 pg mL^−1^	4.1	2.6	1.8	4.4
CV (inter-day, *n* = 9), %	50 pg mL^−1^	–	9.2	8.4	9.0
	750 pg mL^−1^	7.6	3.0	6.4	3.8
	2500 pg mL^−1^	4.7	4.8	5.4	8.0
*R*, %	50 pg mL^−1^	–	113.0	112.1	98.9
	750 pg mL^−1^	105.3	100.6	97.8	105.1
	2500 pg mL^−1^	102.3	97.5	95.6	99.0
ME, %	5 ng mL^−1^	− 10.1 to 13.2	− 12.9 to 12.1	− 6.1 to 9.2	2.8 to 10.2
	50 ng mL^−1^	− 0.7 to 5.0	− 0.1 to 8.1	− 4.9 to 7.4	− 1.0 to 6.2
	350 ng mL^−1^	− 0.6 to 7.6	4.1 to 12.1	− 3.0 to 7.5	− 0.9 to 9.4

Calibration graphs ranging from 5.0 to 7500.0 pg mL^−1^ were prepared for all analytes. The following calibration graph equations were obtained for the studied analytes: for acyclovir *y* = 3154.2*x* − 39.4; for amantadine *y* = 140,802.6*x* − 1829.3; for rimantadine *y* = 33,620.2*x* − 455.8; and for oseltamivir *y* = 243,765.2*x* − 2353.2. The obtained correlation coefficients (*r* > 0.997) imply very good linearity was achieved. The limits of detection and quantification of antiviral drugs achieved in this study are much lower for ACV and OS, and slightly higher for AMA and RIM than those reported in a paper, where these four analytes were determined in wastewater (Vergeynst et al. 2015). Based on the analysis of intra-day and inter-day precision values, it can be concluded that the developed method exhibits excellent precision, with a maximum deviation of 9.2%. Additionally, the method has demonstrated satisfactory recovery values, with no values falling below 95.6% or exceeding 113.0% in any instance.

**Fig. 4:**
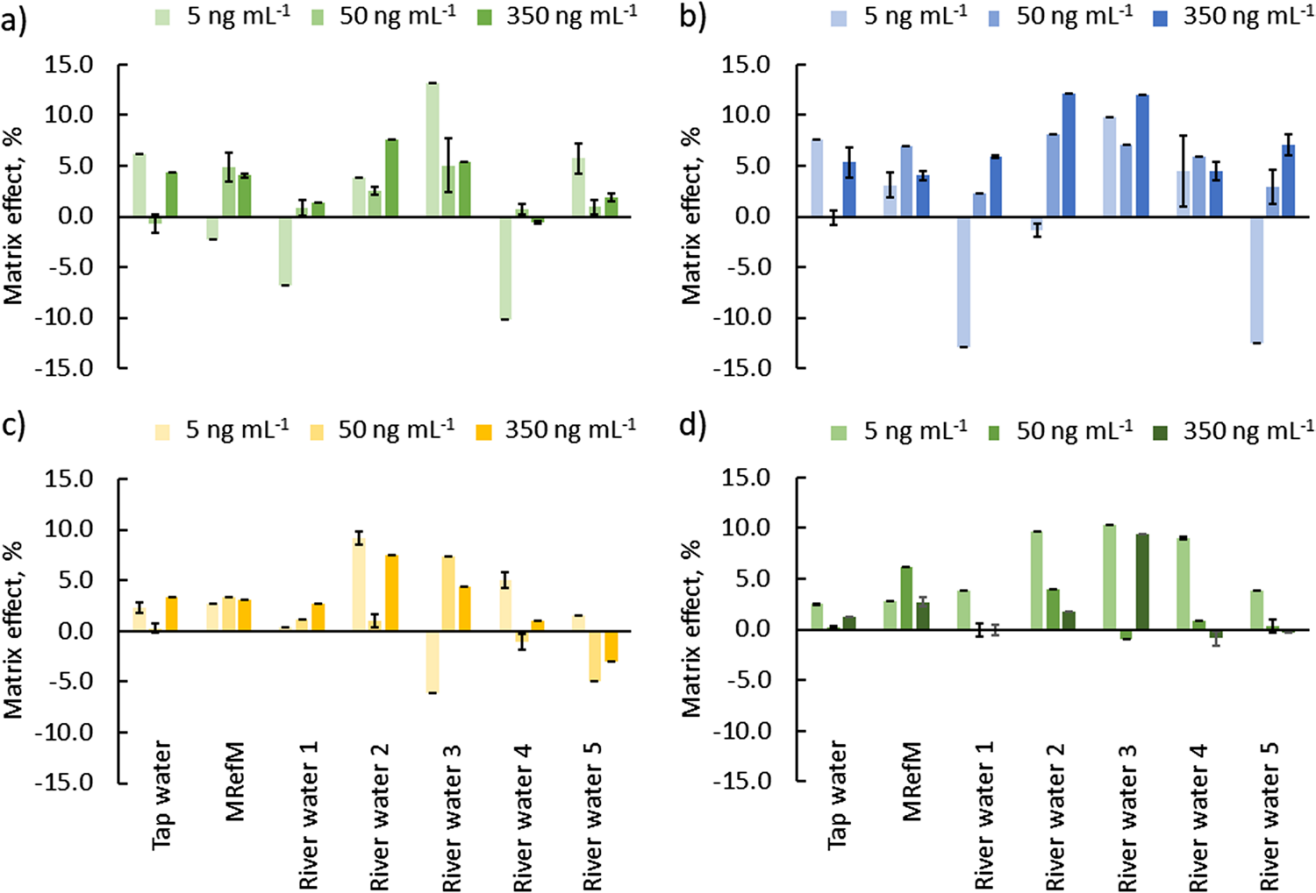
Representation of matrix effects determined for analytes in seven different matrices: **a** acyclovir, **b** amantadine, **c** rimantadine, **d** oseltamivir (bars show the standard deviation; *n* = 3, one sample, three injections)

The matrix effect study was performed to ensure that precision, selectivity, and sensitivity would not be compromised by the samples’ matrix. Establishing this parameter is crucial for developing an analytical method using an UHPLC-MS/MS system. The matrix effect was investigated for seven different water samples: tap water, matrix reference material of drinking water, and five river water samples (two samples from urban areas, samples 1–2, and three samples from the countryside, samples 3–5). The results of the ME study (Fig. 4, Table 3) indicate that the matrix effect was acceptable and well-controlled for all analytes in each examined matrix. None of the cases showed a matrix effect exceeding ± 13.2%. For most quantitative assays, acceptable matrix effect values are assumed to be ± 15%. Therefore, it can be concluded that the matrix effect in the analysed samples is acceptable. Maintaining the matrix effect at such a level was achieved by performing an optimised extraction procedure in the developed FA-SPE system. Furthermore, obtaining low ME values was achieved by selecting the appropriate mass spectrometer mode and ionisation technique. The multiple reaction monitoring mode (MRM) allows the selection of unique precursor-product ion pairs to minimise interference from matrix components. ESI ionisation provides favourable ionisation of the analyte and reduces matrix ionisation.

Regarding the analytical parameters of the developed method and others reported in the literature, in this study, the LOQ values for oseltamivir were higher or comparable to those found in the literature ranging from 0.2 to 12 pg mL^−1^ (Ghosh et al. 2010; Prasse et al. 2010; Azuma et al. 2012). The values for amantadine were higher compared to the value of 0.2 pg mL^−1^ found in the paper (Azuma et al. 2012). As far as the value of LOQ for acyclovir is concerned, it was also higher than those found in the literature, 1 and 6 pg mL^−1^ (Prasse et al. 2010; Peng et al. 2014). However, no article was found in the literature reporting analytical parameters for the method for determining rimantadine in surface water (LOQ 27.3 pg mL^−1^, precision 8.4%, recovery 102–110% in this study). Moreover, the developed method demonstrated good intra-day precision of less than 8.3% and inter-day precision of less than 9.2% for all analytes, which is comparable to the reported literature values (less than 7.4%) (Ghosh et al. 2010; Azuma et al. 2012) but for some methods, precision was not reported (Prasse et al. 2010). Regarding the recovery, the values obtained for acyclovir (102–106%) were comparable to those reported in the literature (100–108%) (Prasse et al. 2010; Peng et al. 2014) and much better for oseltamivir (95–111% this study and 58–128% reported in the literature) (Ghosh et al. 2010; Prasse et al. 2010; Azuma et al. 2012) and for amantadine (97–105% this study and 74–75%) (Azuma et al. 2012) demonstrating the reliability and robustness of our analytical procedure.

### Method application − analysis of water samples

To verify the suitability of the developed method, the developed FA-SPE system with an UHPLC-MS/MS was used to analyse real samples − tap water, matrix reference material of drinking water, and five river water samples. Both unspiked and spiked samples were analysed. The obtained results are summarised in Table 4.

**Table 4:** Results of the determination of selected analytes in water samples

	Exp. conc., pg mL^−1^	Acyclovir			Amantadine			Rimantadine			Oseltamivir		
		Det. conc., pg mL^−1^	*R*, %	CV (*n* = 3), %	Det. conc., pg mL^−1^	*R*, %	CV (*n* = 3), %	Det. conc., pg mL^−1^	*R*, %	CV (*n* = 3), %	Det. conc., pg mL^−1^	*R*, %	CV (*n* = 3), %
Tap water	0.0^a^	< LOD	-	-	< LOD	-	-	< LOD	-	-	< LOD	-	-
	50.0	< LOD	-	-	56.5	128.1	5.2	56.1	121.1	7.1	49.5	98.9	8.3
	750.0	790.1	105.3	4.4	754.4	100.6	3.1	733.5	97.8	2.7	788.3	105.1	3.2
	2500.0	2556.4	102.3	4.1	2437.1	97.5	2.6	2391.0	95.64	1.8	2474.9	99.0	4.4
Drinking water (MRefM)	0.0^a^	< LOD	-	-	< LOD	-	-	< LOD	-	-	< LOD	-	-
	50.0	< LOD	-	-	62.4	124.7	3.3	62.7	125.3	5.4	47.5	95.0	5.0
	750.0	701.3	93.5	6.3	724.1	96.5	7.7	721.4	96.2	1.6	746.1	99.5	5.2
	2500.0	2159.9	86.4	7.6	2475.9	99.0	6.8	2227.3	89.1	5.1	2250.0	90.0	2.2
River water 1	0.0^a^	< LOD	-	-	< LOD	-	-	< LOD	-	-	< LOD	-	-
	50.0	< LOD	-	-	57.8	115.7	4.9	65.8	131.5	5.3	51.3	102.6	4.7
	750.0	725.4	96.7	5.7	75.25	100.3	2.2	715.2	95.4	6.0	729.9	97.3	4.3
	2500.0	2397.3	95.9	5.2	2505.7	100.2	1.5	2478.3	99.1	3.0	2630.4	105.2	4.2
River water 2	0.0^a^	< LOD	-	-	< LOD	-	-	< LOD	-	-	< LOD	-	-
	50.0	< LOD	-	-	63.5	126.9	4.1	52.3	106.6	5.5	36.3	72.6	7.4
	750.0	761.7	101.6	3.2	764.8	102.0	2.1	743.0	88.4	5.2	736.6	98.2	2.5
	2500.0	2307.8	92.3	5.4	2419.4	96.8	5.3	2378.5	98.7	7.6	2358.6	94.3	7.0
River water 3	0.0^a^	< LOD	-	-	< LOD	-	-	< LOD	-	-	< LOD	-	-
	50.0	< LOD	-	-	52.9	105.8	5.1	53.3	101.7	3.6	60.7	112.5	5.7
	750.0	728.5	97.1	3.1	734.3	97.9	8.2	663.4	99.2	3.4	692.1	103.9	5.8
	2500.0	2595.9	103.8	6.7	2559.3	102.4	6.0	2466.7	98.4	8.0	2369.1	102.4	6.4
River water 4	0.0^a^	< LOD	-	-	< LOD	-	-	< LOD	-	-	< LOD	-	-
	50.0	< LOD	-	-	56.1	112.1	7.1	50.9	115.1	9.9	56.3	94.6	3.2
	750.0	768.1	102.4	2.0	721.3	96.2	6.3	743.8	108.5	6.2	779.5	100.5	1.6
	2500.0	2425.8	97.0	5.5	2374.0	95.0	4.4	2460.1	86.5	6.7	2558.8	94.9	3.5
River water 5	0.0^a^	< LOD	-	-	40.1	-	3.9	< LOD	-	-	< LOD	-	-
	50.0	< LOD	-	-	83.5	86.9	2.7	57.6	106.6	7.1	47.3	94.6	7.2
	750.0	755.5	100.7	7.9	760.1	96.0	7.8	813.7	88.4	7.3	753.7	100.5	4.1
	2500.0	2459.3	98.4	7.0	2505.2	98.6	2.4	2162.8	98.7	2.5	2372.7	94.9	4.6

Exp. conc. − expected concentration of the analyte; Det. conc. − determined concentration of the analyte

< *LOD* − below detection limit

^a^Unspiked sample

According to Table 4, amantadine was a drug that was determined in only one river water sample with good precision (40.1 pg mL^−1^, CV 3.9%). The analysis of the other water samples did not reveal the presence of any of the analysed drugs at detectable levels. Several papers have been published in the literature describing the detection of antiviral drugs in river waters in different parts of the world. There have been reports published on finding oseltamivir and acyclovir in surface (river) water. In China, acyclovir was detected in water from the Pearl River at a concentration of 113 pg mL^−1^ (Peng et al. 2014), while during flu season, oseltamivir was found in another area of the country at a concentration of 288 pg mL^−1^ (Söderström et al. 2009; Azuma et al. 2012). In Germany, these drugs were also detected in rivers at concentrations ranging from 2.2 to 190 pg mL^−1^ for acyclovir and from 0.6 to 15 pg mL^−1^ for oseltamivir (Prasse et al. 2010). There are no reports of detecting amantadine and rimantadine in river water; however, reports on the undertaken attempts to determine these drugs in wastewater samples are known. Amantadine was found at concentrations ranging from 50 to 1000 pg mL^−1^, while rimantadine was not detected in the wastewater samples tested (Vergeynst et al. 2015). In Japan, amantadine was detected in wastewater samples at similar levels of 200 to 600 pg mL^−1^ (Ghosh et al. 2010).

To study the analytical suitability of the developed method, it was decided to prepare spiked samples. To this aim, drug samples were prepared in the tested water samples at three concentration levels. The analytical results are presented in Table 4. For ACV, results were obtained at two concentration levels, as the lowest prepared concentration was far below the LOQ (329.8 pg mL^−1^). Nevertheless, the obtained results were good, as the recovery values for ACV ranged from 86.4 to 105.3% and the precision of the determinations did not exceed 7.9%. Satisfactory results were also achieved for the other drugs − amantadine, rimantadine, and oseltamivir. Within each of the river samples, the recovery value for AMA was 86.9–128.1% (CV ≤ 8.2%); for RIM, the recovery value was 86.5–131.5% (CV ≤ 9.9%); and for OS, the recovery value was 72.6–112.5% (CV ≤ 8.3%). Considering the possible concentration ranges of the analysed drugs in which they can be detected in water (Peng et al. 2014; Azuma et al. 2012; Söderström et al. 2009; Prasse et al. 2010; Vergeynst et al. 2015; Ghosh et al. 2010), the obtained results confirmed the applicability of the developed FA-SPE system with the UHPLC-MS/MS method for the preparation and determination of acyclovir, amantadine, rimantadine, and oseltamivir in water samples.

### Greenness evaluation

The developed analytical procedure for determining antiviral drugs in surface waters was assessed for environmental impact (greenness evaluation). More specifically, the sample preparation step was assessed, as this is the main factor distinguishing the method proposed in this paper from those available in the literature. For this purpose, the developed FA-SPE procedure was evaluated and subsequently compared with five other reference sample preparation methods. It is essential to mention that during the literature review, no publication was found where the authors simultaneously determined all of the analytes selected in this work (acyclovir, amantadine, rimantadine, and oseltamivir). Therefore, during the selection of methods to perform the greenness evaluation, it was decided that only papers where at least one of the analytes, oseltamivir, was determined in surface waters would be considered. The authors of every of selected publication provide procedures for the determination of drugs (among others or solely oseltamivir) in surface waters using solid phase extraction and liquid chromatography-mass spectrometry. Based on the literature review, it was noted that none of the found papers reported using a sample preparation and analysis method other than those mentioned above (SPE and LC-MS).

**Fig. 5:**
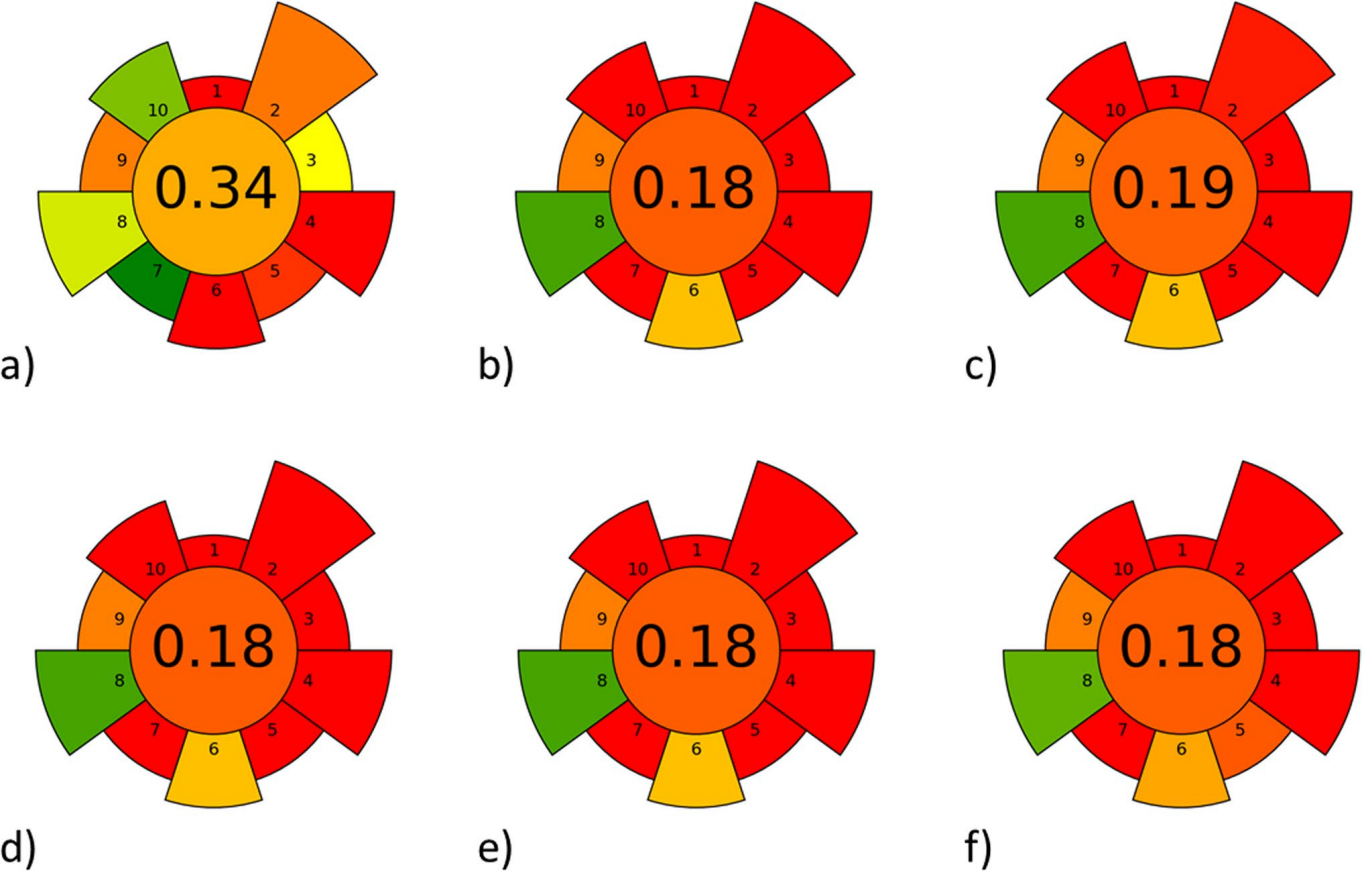
Results of AGREEprep greenness evaluation of six analytical procedures for determination of oseltamivir in surface waters: **a** Method 1, proposed procedure; **b** Method 2 (Ghosh et al. 2010); **c** Method 3 (Söderström et al. 2009); **d** Method 4 (Prasse et al. 2010); **e** Method 5 (Takanami et al. 2012); **f** Method 6 (Azuma et al. 2017); 1–10 categories of evaluation (details in Table S1)

As mentioned previously, many approaches have been developed to perform an analytical method evaluation. Within this work, it was decided to evaluate the greenness of the sample preparation methods using the Analytical Greenness Metric for Sample Preparation (AGREEprep) (Wojnowski et al. 2022). It is worth noting that the default weights for the individual categories were maintained − to avoid introducing any favouritism. Results of the greenness evaluation of six analytical procedures for the determination of oseltamivir in surface waters are shown in Fig. 5, while the data used to evaluate and compare the method are provided in Table S1 (Supplementary materials).

As shown in Fig. 5, all of the approaches employed for the preparation of surface water samples for analysis for the determination of antiviral drugs have produced results that are distant from satisfactory in terms of environmental impact. Such a situation is mainly caused by the application of solid phase extraction itself, which requires the consumption of large volumes of, often hazardous, reagents; it is time-consuming and multistep and operator safety is limited. The FA-SPE procedure proposed in this work received a significantly higher total score in the greenness metric than the others because sample preparation was simplified. The FA-SPE method has been evaluated based on different categories of greenness (Table S1). It has proven to be more effective in categories, which include evaluation parameters such as hazardous material volume (2); sustainability, renewability, and reusability of materials (3); size economy of sample (5); integration and automation (27); and operator’s safety (10). However, it remains behind in categories, which relate to sample throughput (6) and energy consumption (8). It should also be noted that in terms of the number of analytes determined in a single run, in this work, four analytes were determined simultaneously, whereas in the other reviewed papers, up to ten compounds were determined.

The developed FA-SPE system allows a fully automated process − all extraction steps are conducted automatically, without the analyst’s involvement, ensuring the analyst’s safety. A further advantage of the developed procedure is the use of extraction columns with a small sorbent mass (30 mg). This solution allows the use of small volumes of reagents and a sample. Such columns are commercially available, so anyone can use them while maintaining constant and reproducible operating conditions. In this study, it was demonstrated that the columns can be used several times (“Testing of the SPE column performance” section) without loss of analytical efficiency. This underscores the system’s potential as a robust and environmentally conscious sample preparation methodology.

## Conclusions

The proposed flexible FA-SPE system, along with a highly sensitive UHPLC-MS/MS technique for chromatographic separation and analyte detection, allowed the development of a methodology for the determination of selected antiviral drugs (acyclovir, amantadine, rimantadine, and oseltamivir) in surface water samples. The proposed UHPLC-MS/MS method allowed obtain low LOD values for analytes and make it a competitive method in terms of analytical performance. The FA-SPE system, developed for automated and integrated extraction, enhances operator safety and minimises human impact on analysis results. This solution ensures high precision and reproducibility, facilitated by automation that eliminates the time-consuming step of solvent evaporation. The system’s design prioritises compactness, manageability, and portability, facilitating sampling and preparation beyond the laboratory for continuous water monitoring. Additionally, the proposed FA-SPE sample preparation method is environmentally friendly compared to alternative methods for determining antiviral drugs in surface waters reported in the literature. The proposed FA-SPE sample preparation approach is also advantageous in terms of environmental impact compared to other sample preparation methods reported in the literature for the determination of selected antiviral drugs in surface waters. The obtained analytical results provide an opportunity for the method to be successfully used for the analysis of other types of samples, e.g. wastewater.

### Supplementary Information

Below is the link to the electronic supplementary material.

Supplementary file1 (DOCX 129 KB)

## Data Availability

The datasets used in this study are available from the corresponding author on reasonable request.
