# Supplementary material for: Flow analysis-solid phase extraction system and UHPLC-MS/MS analytical methodology for the determination of antiviral drugs in surface water
Source: Environ Sci Pollut Res Int. 2024 Jul 30;31(37):49546–59. doi: 10.1007/s11356-024-34466-5 (PMC11324774; doi:10.1007/s11356-024-34466-5)
Supplement: Supplementary file 1 — Supplementary file1 (DOCX 129 KB) [file 11356_2024_34466_MOESM1_ESM.docx]

**SUPPLEMENTARY MATERIALS**

**Flow analysis-solid phase extraction system and UHPLC-MS/MS analytical methodology for the determination of antiviral drugs in surface water**

Karolina Mermer^1,2^*, Emilia Jas^2^, Justyna Paluch^2^, Aneta Woźniakiewicz^2^, Michał Woźniakiewicz^2^, Paweł Miśkowiec^3^, Petr Chocholouš^4^, Hana Sklenářová^4^, Joanna Kozak^2^

^1^Doctoral School of Exact and Natural Sciences, Jagiellonian University, Łojasiewicza 11, 30-348 Kraków, Poland.

^2^Department of Analytical Chemistry, Faculty of Chemistry, Jagiellonian University, Gronostajowa 2, 30-387 Kraków, Poland.

^3^Department of Environmental Chemistry, Faculty of Chemistry, Jagiellonian University, Gronostajowa 2, 30-387 Kraków, Poland.

^4^Department of Analytical Chemistry, Faculty of Pharmacy in Hradec Králové, Charles University, Heyrovského 1203/8, 50003 Hradec Králové, Czech Republic.

*Corresponding author: [karolina.mermer@doctoral.uj.edu.pl](mailto:karolina.mermer@doctoral.uj.edu.pl)


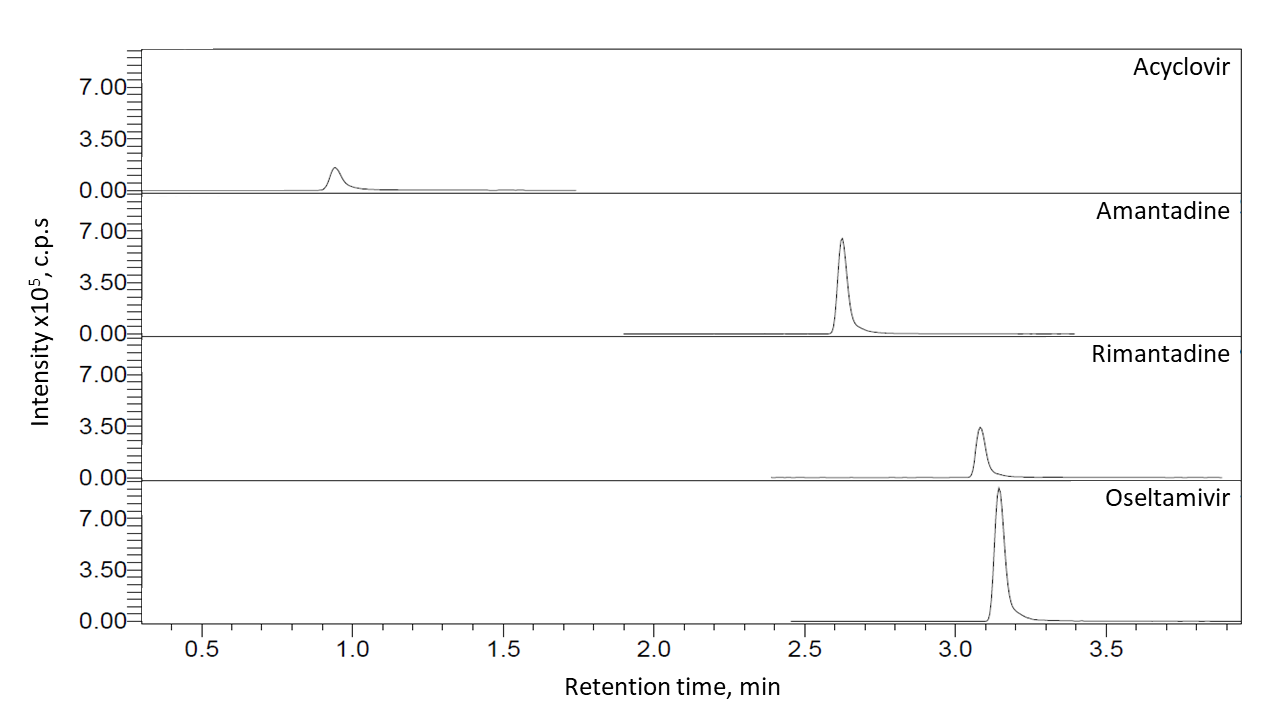


**Fig. S1** The chromatogram obtained during the analysis of a mixed standard solution of the analytes at a concentration of 1 µg/mL by the UHPLC-MS/MS method

**Table S1** The data used for greenness evaluation and comparison of the methods for sample preparation using AGREEprep (Figure 5)

| **Category** | **1** | **2** | **3** | **4** | **5** | **6** | **7** | **8** | **9** | **10** |
| --- | --- | --- | --- | --- | --- | --- | --- | --- | --- | --- |
| **The method** (and reference) | **Sample preparation placement** | **Hazardous materials**, mL | **Sustainability, renewability, and reusability of materials** | **Waste**, mL | **Size economy of sample**, mL | **Sample throughput** (number of samples per h) | **Integration and automation** (number of steps; automation degree) | **Energy consumption** ([Wh] per sample) | **Post-sample preparation configuration for analysis** | **Operator’s safety** (number of hazardous substances and physical threats) |
| **Weight** (default value) | 1 | 5 | 2 | 4 | 2 | 3 | 2 | 4 | 2 | 3 |
| **Method 1** (this paper) | Ex situ | 2 | Materials are not sustainable or renewable but are used several time | 58 | 50 | 1 | 1; fully automated system | 50 | LC, GC with quadrupole detection, etc. | 1* |
| **Method 2**^1^ | Ex situ | ~10* | < 25% of reagents and materials are sustainable or renewable | ~ 311* | 300 | ~ 5* | 6*; manual system | 17* |  | 4* |
| **Method 3**^2^ | Ex situ | ~ 7* |  | ~ 510* | 500 | ~ 5* | 8*; manual system | 17* |  | 5* |
| **Method 4**^3^ | Ex situ | ~ 20* |  | ~ 528* | 500 | ~ 5* | 6*; manual system | 17* |  | 4* |
| **Method 5**^4^ | Ex situ | ~ 17* |  | ~ 135* | 100 | ~ 5* | 6*; manual system | 17* |  | 5* |
| **Method 6**^5^ | Ex situ | ~ 12* |  | ~ 58* | 30 | ~ 4* | 10*; manual system | 21* |  | 5* |

* approximate values of parameters based on information in an article

1 G. C. Ghosh, N. Nakada, N. Yamashita and H. Tanaka, *Environ. Health Perspect.*, 2010, **118**, 103–107.

2 H. Söderström, J. D. Järhult, B. Olsen, R. H. Lindberg, H. Tanaka and J. Fick, *PLoS ONE*, 2009, **4**, e6064.

3 C. Prasse, M. P. Schlüsener, R. Schulz and T. A. Ternes, *Environ. Sci. Technol.*, 2010, **44**, 1728–1735.

4 R. Takanami, H. Ozaki, R. R. Giri, S. Taniguchi and S. Hayashi, *J. Wat. Envir. Tech.*, 2012, **10**, 57–68.

5 T. Azuma, M. Ishida, K. Hisamatsu, A. Yunoki, K. Otomo, M. Kunitou, M. Shimizu, K. Hosomaru, S. Mikata and Y. Mino, *Chemosphere*, 2017, **169**, 550–557.
